# Neddylation inhibitor MLN4924 suppresses cilia formation by modulating AKT1

**DOI:** 10.1007/s13238-019-0614-3

**Published:** 2019-03-09

**Authors:** Hongmei Mao, Zaiming Tang, Hua Li, Bo Sun, Mingjia Tan, Shaohua Fan, Yuan Zhu, Yi Sun

**Affiliations:** 1 The Cancer Institute of the Second Affiliated Hospital, and Institute of Translational Medicine, Zhejiang University School of Medicine, Hangzhou, 310029 China; 2 Division of Radiation and Cancer Biology, Department of Radiation Oncology, University of Michigan, Ann Arbor, MI 48109 USA; 3 Gilbert Family Neurofibromatosis Institute, Center for Cancer and Immunology Research, Children’s Research Institute, Children’s National Medical Center, Washington, DC 20010 USA

**Keywords:** AKT, Cilia, MLN4924, neddylation, siRNA, VHL

## Abstract

The primary cilium is a microtubule-based sensory organelle. The molecular mechanism that regulates ciliary dynamics remains elusive. Here, we report an unexpected finding that MLN4924, a small molecule inhibitor of NEDD8-activating enzyme (NAE), blocks primary ciliary formation by inhibiting synthesis/assembly and promoting disassembly. This is mainly mediated by MLN4924-induced phosphorylation of AKT1 at Ser473 under serum-starved, ciliary-promoting conditions. Indeed, pharmaceutical inhibition (by MK2206) or genetic depletion (via siRNA) of AKT1 rescues MLN4924 effect, indicating its causal role. Interestingly, pAKT1-Ser^473^ activity regulates both ciliary synthesis/assembly and disassembly in a MLN4924 dependent manner, whereas pAKT-Thr^308^ determines the ciliary length in MLN4924-independent but VHL-dependent manner. Finally, MLN4924 inhibits mouse hair regrowth, a process requires ciliogenesis. Collectively, our study demonstrates an unexpected role of a neddylation inhibitor in regulation of ciliogenesis via AKT1, and provides a proof-of-concept for potential utility of MLN4924 in the treatment of human diseases associated with abnormal ciliogenesis.

**Electronic supplementary material:**

The online version of this article (10.1007/s13238-019-0614-3) contains supplementary material, which is available to authorized users.

## INTRODUCTION

The primary cilium (immotile cilium), a microtubule-based organelle, that projects from the apical surface of most eukaryotic cells, has emerged as a key organelle involved in many physiological and developmental processes (Goetz and Anderson, 2010). The primary cilium acts like a cellular ‘antenna’ to sense and respond to the extracellular environment (Ishikawa and Marshall, 2011). Defects in ciliogenesis or ciliary function are responsible for many human ciliopathies, commonly sharing the pathological combinations of kidney cyst, mental retardation, retinal degeneration, polydactyly, diabetes and obesity (Singla and Reiter, 2006; Eggenschwiler and Anderson, 2007). Potential involvement of abnormal primary cilia in tumorigenesis was also implicated, as evidenced by reduced or loss of primary cilia in human cancer cell lines derived from breast carcinoma, melanoma, clear cell renal cell carcinoma (Liu et al., 2018). However, whether ciliary dysfunction is a cause or a consequence of tumorigenesis remains elusive.

Neddylation modification is a process of covalent attachment of NEDD8, a ubiquitin-like peptide, to a lysine residue of a given protein substrate, catalyzed by NEDD8-activating enzyme (NAE, E1), NEDD8-conjugating enzyme (E2), and NEDD8 ligase (E3) (Zhao et al., 2014). MLN4924, also known as pevonedistat, is a specific small molecule inhibitor of NAE (Soucy et al., 2009), currently in clinical trials for the treatment of cancer (Nawrocki et al., 2012; Zhou et al., 2018). MLN4924 binds to NAE to form a covalent NEDD8-MLN4924 adduct that cannot be further utilized in subsequent intraenzyme reactions, thus blocking the entire neddylation modification (Brownell et al., 2010). Extensive preclinical studies have shown that MLN4924 induces growth arrest, apoptosis, senescence and autophagy in a variety of human cancer cell lines both *in vitro* cell culture setting and *in vivo* xenograft models (Zhao et al., 2014; Zhou et al., 2018). MLN4924 also showed to block angiogenesis and sensitize cancer cells to chemo- and radiation therapies (Zhou et al., 2018). Whether and how MLN4924 regulates cilium formation is completely unknown.

AKT, also known as protein kinase B (PKB), is a family of pleckstrin homology PH domain containing serine threonine kinases with three members AKT1, AKT2, and AKT3, encoded by paralogous genes. Three members are closely related, but functionally distinct (Wang et al., 2017). AKT1/PKB is activated in a phosphoinositide 3-kinase (PI3K)-dependent manner (Sarbassov et al., 2005). Full activation of AKT/PKB requires the phosphorylation at both Thr308 and Ser473. Phosphoinositide-dependent kinase 1 (PDK1) is responsible for the phosphorylation at Thr308, whereas phosphorylation at Ser473 is fulfilled by PDK2 which includes mTOR complex 2 (mTORC2) and integrin-linked kinase (ILK) (Persad et al., 2001). The PI3K/AKT axis is one of the most important intracellular signaling pathways that regulates many important biological processes including proliferation, autophagy, apoptosis and differentiation, and its deregulation is actively involved in many human diseases, including cancer (Persad et al., 2001; Fresno Vara et al., 2004; Zhang et al., 2012). However, potential involvement of PI3K/AKT in ciliogenesis is much less studied. One study showed that inhibition of AKT1/2 suppressed both ciliogenesis and cilia length (Suizu et al., 2016), whereas another study, in contrast, reported that increased PI3K/AKT signaling promoted cilia loss without determining the effect on cilia length (Conduit et al., 2017). Thus, whether and how AKT1 and its family members regulate ciliogenesis remain elusive.

The VHL (von Hippel-Lindau) tumor-suppressor gene is responsible for an autosomal-dominant inherited tumor syndrome that manifests as hemangioblastomas of the retina and central nervous system combined with renal clear cell carcinoma and pheochromocytoma (Richards, 2001). The best characterized function of pVHL (the product of *VHL* gene) is to act as a substrate recognition component of Cullin-2 E3 ligase for targeted ubiquitylation and degradation of HIF-1α (hypoxia inducible factor-1α) in an oxygen-dependent manner (Kaelin, 2008). In addition, pVHL interacts with many other proteins to regulate a variety of biological processes, including microtubule dynamics, cell proliferations, extracellular matrix deposition and primary cilia maintenance (Frew and Krek, 2007). While it was reported that pVHL positively regulates primary cilia through both HIF-1-dependent and -independent mechanisms (Kuehn et al., 2007), its potential involvement in the regulation of ciliogenesis by neddylation is previously unknown.

In the present study, we showed that MLN4924, a small molecule inhibitor of protein neddylation, significantly suppressed cilia assembly and promoted cilia disassembly. Mechanistically, the MLN4924 effect appears to be mediated by activation of pAKT-Ser^473^ and can be blocked by AKT1 inhibitor or siRNA-based AKT1 silencing. Furthermore, we found that pAKT-Thr^308^ appear to negatively regulates cilia length in a VHL-dependent, but MLN-4924 independent manner. Finally, we found that hair regrowth, a process dependent of primary cilia can be inhibited by MLN4924. Collectively, our study provides the proof-of-concept evidence that MLN4924 may have utility in the treatment of human diseases associated with abnormal cilia outgrowth seen in some types of cancer, such as adenocarcinoma of the lung and colon, follicular lymphoma and a subtype of medulloblastoma (Han et al., 2009; Yasar et al., 2017).

## RESULTS

### MLN4924 blocks ciliogenesis by inhibiting initiation and promoting disassembly

**Figure 1:**
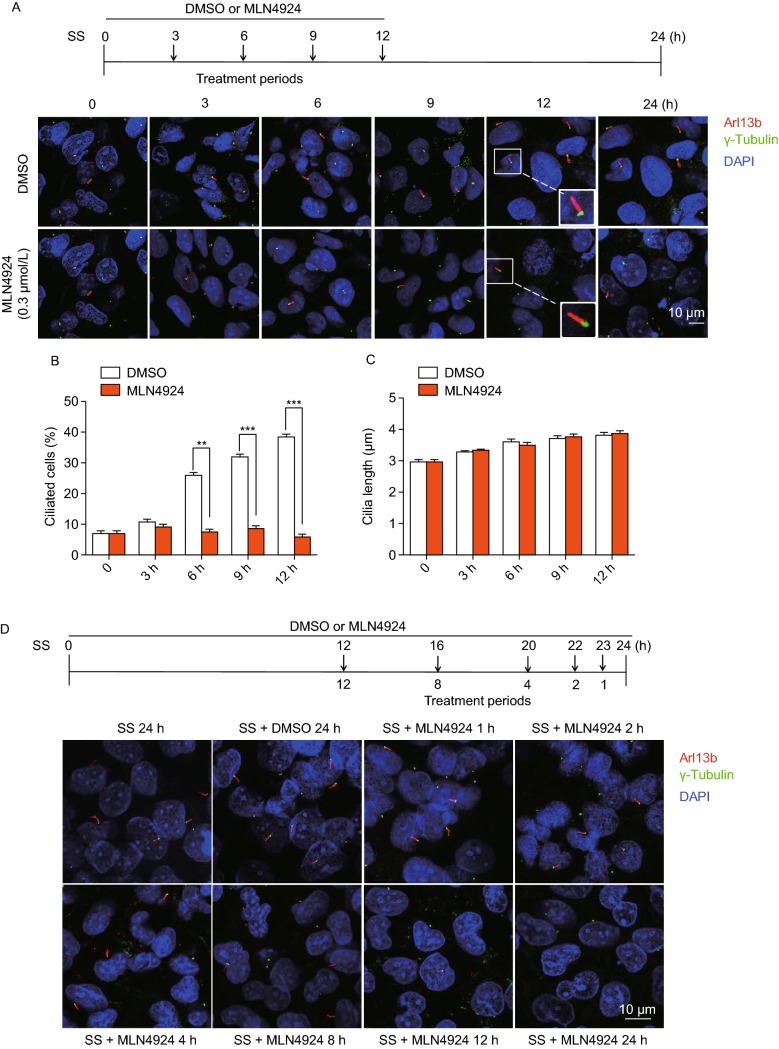

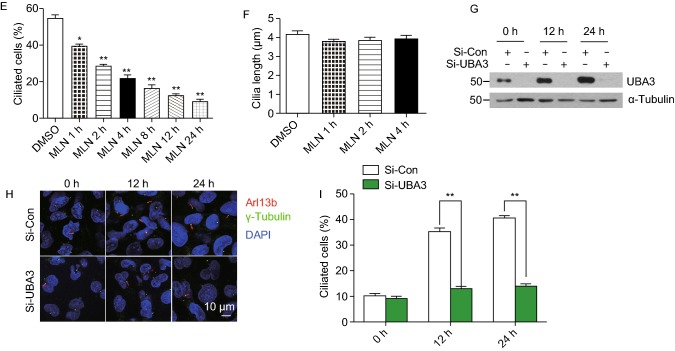
**MLN4924 blocks ciliogenesis by inhibiting initiation and promoting disassembly**. (A–C) Effect of MLN4924 on cilia initiation: BEAS2B cells were treated with DMSO or MLN4924 (0.3 μmol/L) at the beginning of serum starvation for indicated periods of time (A, top panel), followed by immunofluorescence staining with anti-Arl13b and γ-tubulin (γ-tub) antibodies, and photography (A, bottom panel). Percentage of ciliated cells were counted (B) or cilia length measured (C), and the results plotted. Shown is mean ± SEM from three independent experiments. **P < 0.01, ***P < 0.001. (D–F) Effect of MLN4924 on cilia disassembly: BEAS2B cells were treated with MLN4924 (0.3 μmol/L) for entire 24 h, or at 12 h post serum starvation for indicated time periods (D, top panel), followed by immunofluorescence and photography (D, bottom panel). Percentage of ciliated cells were counted (E) or cilia length measured (F), and the results plotted. Shown is mean ± SEM from three independent experiments. **P < 0.01. (G–I) UBA3 knockdown significantly inhibits cilia formation: BEAS2B cells were transfected with scrambled control siRNA or siUBA3 under serum starved condition for indicated periods of time, followed by western blotting with indicated Ab (G) or immunofluoresence staining of cilia and photography (H). Percentage of ciliated cells were counted (I), and the results plotted. Shown is mean ± SEM from three independent experiments. **P < 0.01

Previous studies have shown that ubiquitylation regulates cilia assembly and disassembly (Hossain and Tsang, 2018), and several ciliary-associated proteins such as CP110 (D’Angiolella et al., 2010) Aurora A (Kwon et al., 2012) are substrates of Cullin-RING ligases (CRLs), suggesting that CRL may regulate ciliogenesis. To pursue this, we used MLN4924, a small molecule that specifically inhibits NAE to block the entire neddylation process, thus indirectly inhibiting ligase activity of CRLs, which requires cullin neddylation (Deshaies, 1999). Cilia assembly was initiated in immortalized human bronchial epithelium BEAS2B cells by serum starvation. Arl13b and acetylated tubulin (Ac-tub) were used as the markers for primary cilia, whereas γ-tubulin was used as the marker for centrioles (Fig. S1A). MLN4924 treatment, along with the DMSO control, was conducted at the beginning of serum starvation for various time periods up to 12 h (Fig. 1A, top panel). In DMSO control group, percentage of ciliated cells increased, as starvation period prolonged. MLN4924 treatment caused a significant inhibition of cilia initiation, starting at 6 h and extended up to 12 h (Fig. 1A and 1B). However, MLN4924 had no effect on the cilia length (Fig. 1C). Similar results were observed in human retinal pigment epithelial (RPE1) cells (Fig. S1B and S1C). We confirmed that the concentration used for MLN4924 (0.3 μmol/L) effectively blocked cullin neddylation within 1 h of treatment (Fig. S1D and S1E), was not cytotoxic (20-fold less than IC_50_) (Fig. S1F), and with no obvious effect on cells population stayed at the G_0_/G_1_ phase (Fig. S1G).

We next determined potential effect of MLN4924 on cilia disassembly. In a window of 24 h post serum starvation, cells were treated with MLN4924 for entire 24 h, or at 12 h post serum starvation, when cilia were formed, for various time periods (from 1 to 12 h), (Fig. 1D, top panel). A time-dependent reduction in percentage of ciliated cells were observed which were statistically significant in all time points tested, indicating that MLN4924 also effectively accelerated cilia disassembly (Fig. 1D and 1E). Again, MLN4924 had no effect on cilia length and cell population at the G_0_/G_1_ phase (Fig. S1H). The similar results were observed in IMCD3 cells, a murine inner medullary collecting duct line (Fig. S1I and S1J). Finally, we used genetic approach via siRNA-based silencing of Uba3/NAEβ, the catalytic subunit of NAE to which MLN4924 binds (Brownell et al., 2010), and showed that Uba3/NAEβ depletion significantly inhibits cilia formation (Fig. 1G–I). Collectively, these results clearly demonstrated that neddylation inhibitor MLN4924 suppresses cilia formation by inhibiting assembly and promoting disassembly, and this process is independent of cell cycle arrest at the G_0_/G_1_ phase.

### AKT activation plays a causal role in MLN4924-induced suppression of cilia initiation

To elucidate the mechanism by which MLN4924 inhibits ciliogenesis, we first examined potential involvement of CP110 and Cep97, two well-studied centriolar proteins which suppressed cilia assembly (Spektor et al., 2007). Also CP110 was previously reported to be a substrate of SCF/CRL1^cyclinF^ E3 ligase (D’Angiolella et al., 2010), thus likely being subjected to MLN4924 regulation. In BEAS2B cells, serum starvation (DMSO group) increased the levels of CP110, which is not affected by MLN4924 treatment. On the other hand, two forms of Cep97 were detected, and serum starvation induced conversion of the slow-migrating form to the fast-migrating form. Interestingly, MLN4924 blocked this conversion to maintain Cep97 in the duplet status, although it is unclear whether Cep97 activity is affected (Fig. S2A). Importantly, depletion of CP110 (Fig. S2B) or Cep97 (Fig. S2C) reduced ciliated cell population, but had no effect on MLN4924 inhibition of cilia assembly (Fig. S2D and S2E). Our observations that serum starvation induced CP110, and CP110 knockdown reduced cilia assembly suggested that CP110 acts as a promotor of ciliogenesis in BEAS2B cells. It is worth noting that two recent studies showed that CP110 can act as either an inhibitor or a promoter of ciliogenesis (Walentek et al., 2016; Yadav et al., 2016). It was also reported that Aurora A was involved in the process of cilia disassembly (Pugacheva et al., 2007). We, therefore, determined potential effect of MLN4924 under cilia disassembly condition in BEAS2B cells. MLN4924 had no effect on the levels of total or phospho-Aurora, nor on the distribution of pAurora-positive cells at centriole (Fig. S2F and S2G). Taken together, none of common cilia regulatory proteins, including CP110, Cep97 and Aurora A, was involved in MLN4924 effect on cilia initiation or disassembly under our experimental settings.

**Figure 2:**
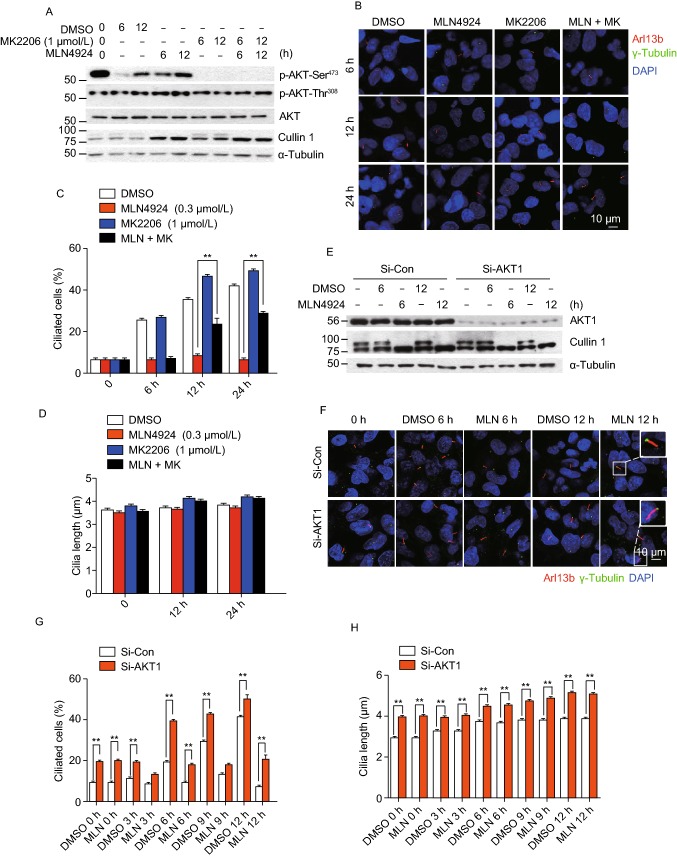

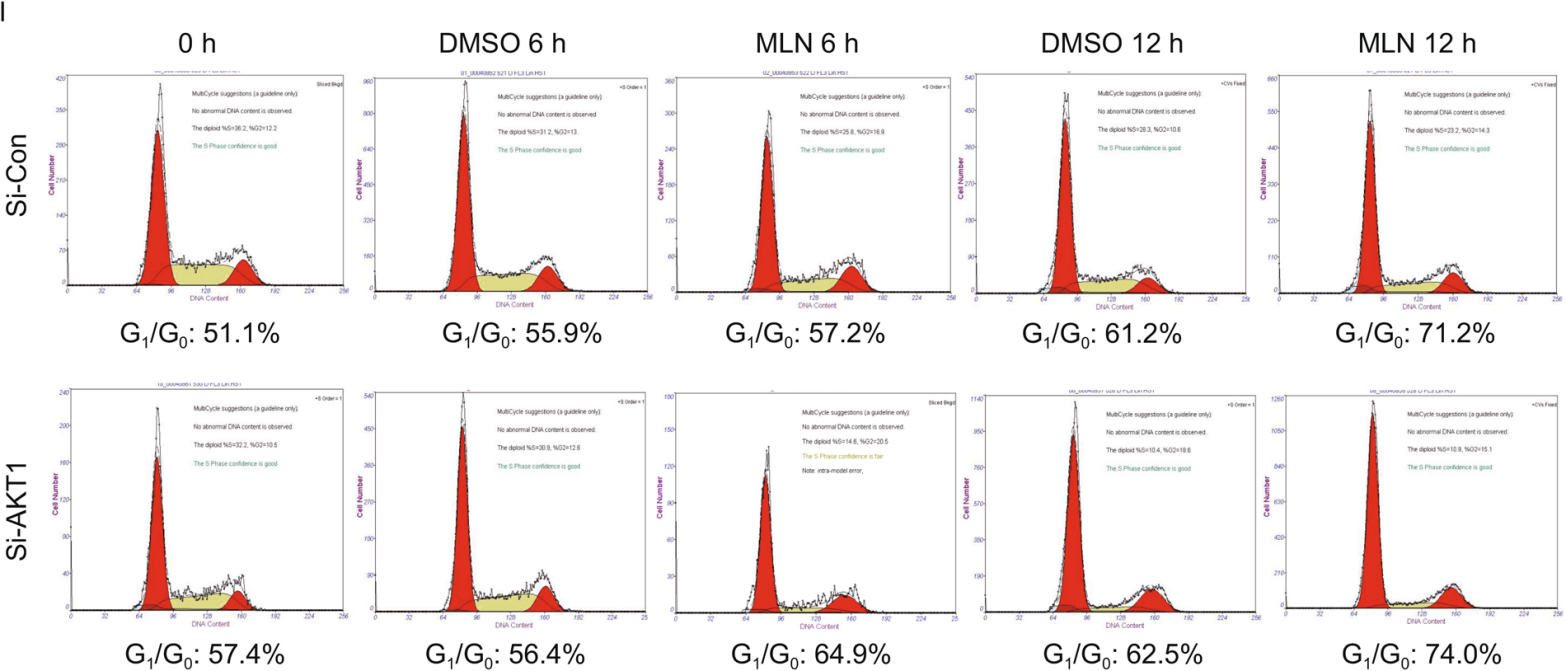
**AKT activation plays a causal role in MLN4924-induced suppression of cilia initiation**. (A–D) Effect of AKT1 activation on ciliogenesis: BEAS2B cells were cultured in normal serum containing medium (lane 1), then switched to serum starved condition (lanes 2**–**9) and treated with MLN4924 (0.3 μmol/L) or MK2206 (1 μmol/L) alone or in combination indicated periods of time, followed by western blotting with indicated Ab (A) or by immunofluorescence staining with anti-Arl13b and γ-tubulin (γ-tub) antibodies, and photography (B). Percentage of ciliated cells were counted (C) or cilia length measured (D), and the results plotted. Shown is mean ± SEM from three independent experiments. **P < 0.01. (E–H) Effect of AKT1 knockdown on cilia formation: BEAS2B cells were transfected with scrambled control siRNA or siAKT1, treated with DMSO or MLN4924 (0.3 μmol/L) under serum starved condition for indicated periods of time, and Western blotting with indicated Abs (E) or immunofluoresence staining of cilia and photography (F). Percentage of ciliated cells were counted (G) or cilia length measured (H) and the results plotted. Shown is mean ± SEM from three independent experiments. **P < 0.01. (I**)** AKT1 knockdown has no effect on the G0/G1 phase: BEAS2B cells were transfected with scrambled control siRNA or siAKT1, treated with DMSO or MLN4924 (0.3 μmol/L) treatment under serum starved condition for cilia initiation, followed by FACS analysis. Shown are percentage of cells at the G_0_/G_1_

We recently showed that MLN4924 could activate AKT1 upon EGF addition to serum-starved cells via inducing epidermal growth factor receptor (EGFR) dimerization (Zhou et al., 2016), and two studies reported that EGFR inhibitor could maintain ciliogenesis, and prevented smoke mediated ciliary loss in airways (Khan et al., 2016; Valencia-Gattas et al., 2016), suggesting that the EGFR/AKT1 signal may mediate MLN4924 effect on ciliogenesis. We, therefore, determined whether MLN4924 blockage of ciliogenesis can be rescued by small molecule inhibitors of EGFR or AKT. In BEAS2B cells, MLN4924 activated EGFR, whereas EGFR inhibitor Erlotinib effectively blocked EGFR phosphorylation and completely rescued MLN4924 inhibitory effect on cilia formation in a cilia disassembly assay (Fig. S2H and S2I). We next focused on AKT1, a downstream target of EGFR signal and found that MLN4924 effectively induced AKT1 phosphorylation at Ser473 without much effect on Thr308 (Fig. 2A, compared lanes 2&3 vs. 4&5). Importantly, inactivation of pAKT1-Ser^473^ by its inhibitor MK2206 at 1 μmol/L, which had minor, if any, effect on pAKT1-Thr^308^ (Fig. 2A), partially rescued MLN4924 inhibitory effect on cilia assembly in time-dependent manners, while MK2206 alone had no effect (Fig. 2B and 2C). Neither compound alone nor their combination had any effect on ciliary length (Fig. 2D).

We further determined causal role of AKT1 via siRNA-based knockdown (Fig. 2E), and found that AKT1 knockdown (siAKT1) significantly stimulated cilia assembly at all-time points tested, and rescued MLN4924 inhibitory effect at 6 h time points and thereafter (Fig. 2F and 2G). Interestingly, siAKT1 significantly stimulated cilia growth, as evidenced by extended ciliary length in a manner independent of MLN4924 (Fig. 2H). Finally, we showed that compared to siCont, siAKT1 had no effect on G_0_/G_1_ population (Fig. 2I), excluding the possibility that siAKT1 regulated cilia assembly through altering cell cycle progression. Collectively, it appears that MLN4924-induced AKT1 activation via Ser473 phosphorylation is responsible at least in part for its inhibitory effect on ciliary assembly, and AKT1 protein amount rather than its pAKT1-Ser^473^ kinase activity regulates ciliary length in a MLN4924-independent manner.

### AKT1 activation also plays a causal role in MLN4924-promoted cilia disassembly

**Figure 3:**
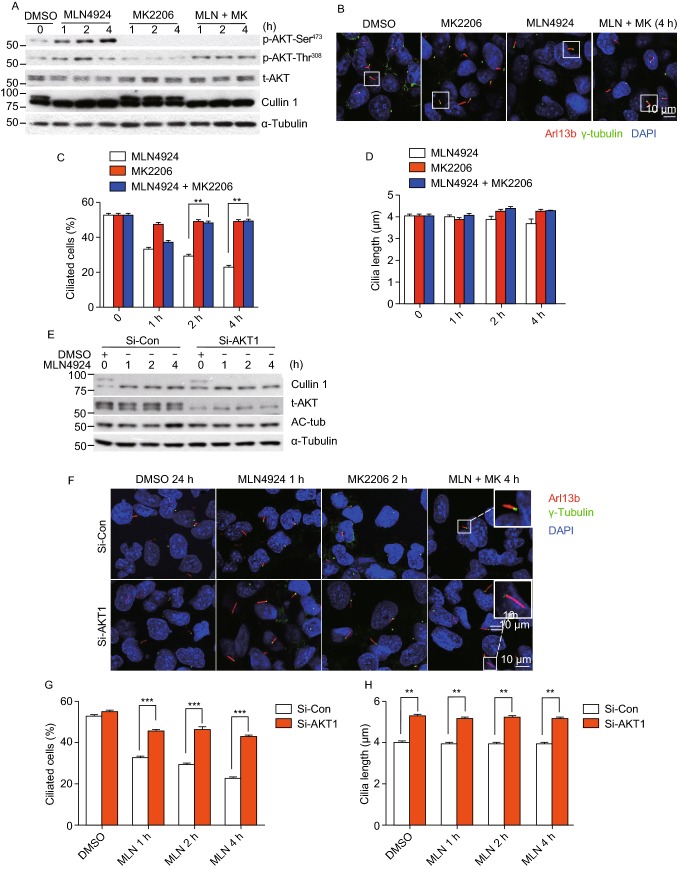
**AKT activation plays a causal role in MLN4924-promoted cilia disassembly**. (A–D) Effect of AKT1 activation on cilia disassembly: BEAS2B cells were treated with MLN4924 (0.3 μmol/L) or MK2206 (1 μmol/L) alone or in combination under serum starved condition for cilia disassembly for indicated periods of time, followed by Western blotting with indicated Abs (A), or by immunofluorescence staining, and photography (B). Percentage of ciliated cells were counted (C) or cilia length measured (D), the results plotted. Shown is mean ± SEM from three independent experiments. **P < 0.01. (E–H) Effect of AKT1 knockdown: BEAS2B cells were transfected with scrambled control siRNA or siAKT1, then treated with DMSO or MLN4924 (0.3 μmol/L) under cilia disassembly condition for indicated periods of time, followed by western blotting with indicated Ab (E) or immunofluoresence staining of cilia and photography (F). Percentage of ciliated cells were counted (G), cilia length measured (H), and the results plotted. Shown is mean ± SEM from three independent experiments. **P < 0.01, ***P < 0.001

Compared to cilia assembly, mechanistic details of cilia disassembly are much less understood in normal or pathological conditions (Cao et al., 2009; Liang et al., 2016). To further investigate possible role of AKT1 in cilia disassembly, we employed cilia disassembly assay condition (Fig. 1D) and added MLN4924 or MK2206, alone or in combination into serum-free medium after cilia formation. Again, MLN4924 increased the levels of pAKT1-Ser^473^, whereas MK2206 effectively inhibited it. On the other hand, MLN4924 or MK2206 only moderately increased or decreased the levels of AKT1-Thr^308^, respectively (Fig. 3A). Although MK2206 itself had no effect on cilia disassembly, it fully rescued the disassembly-promoting effect of MLN4924 (Fig. 3B and 3C). Again, both drugs had no effect on cilia length (Fig. 3D).

We further used a siRNA-based knockdown approach, and found siAKT1 (Fig. 3E) fully rescued MLN4924 disassembly-promoting effect (Fig. 3F and 3G), and again extended cilia length in a MLN4924-independent manner (Fig. 3H). Similar siAKT1 rescuing effect was observed in RPE1 cells (Fig. S3A–C) without affecting the G_1_/G_0_ population (Fig. S3D).

It was reported that RICTOR is mainly responsible for the AKT1 phosphorylation at Ser473 (Cicenas, 2008). We, therefore, determined potential involvement of RICTOR in MLN4924 activation in pAKT-Ser^473^. We found that MLN4924, at different incubation time or doses, did not increase RICTOR protein levels under both cilia assembly and disassembly conditions (Fig. S3E), and RICTOR knockdown (siRICTOR) partially blocked MLN4924-induced pAKT1-Ser^473^ activatation, but did not rescue cilia initiation-inhibiting effect of MLN4924 (Fig. S3F&G), suggesting a requirement for complete pAKT1-Ser^473^ inactivatation, as shown in Fig. 2A–C. Taken together, it appears that MLN4924 activated pAKT-Ser^473^ to block cilia initiation or promote cilia disassembly in a RICTOR-independent manner, whereas AKT amount is responsible for cilia length under both assembly and disassembly conditions independent of MLN4924.

### AKT1, but not its family members, AKT2 or AKT3, regulates ciliogenesis and disassembly

**Figure 4:**
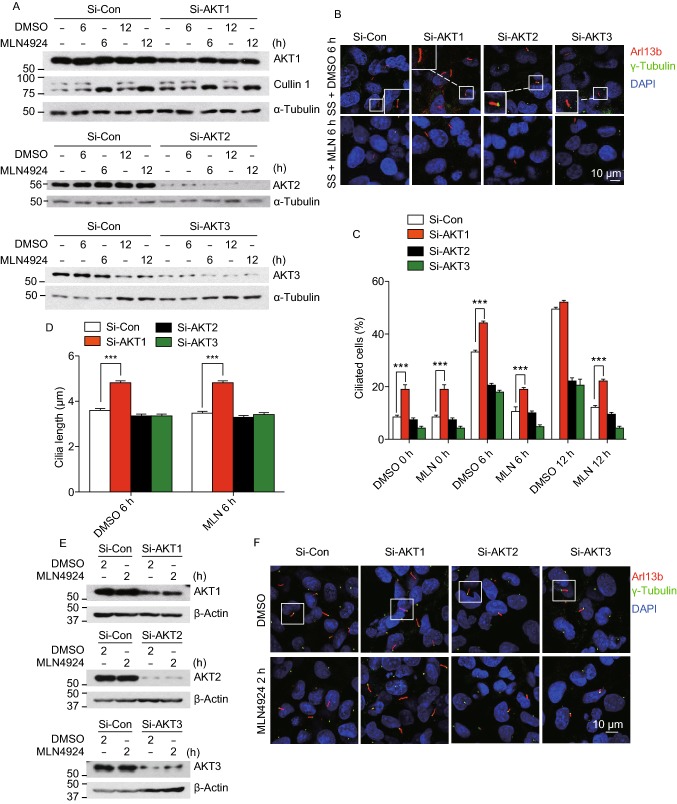

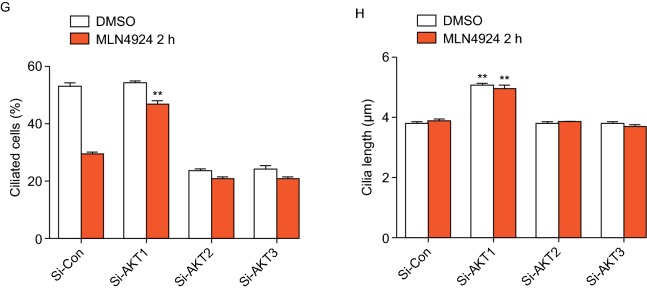
**AKT1, but not its family members, AKT2 or AKT3, regulates ciliogenesis and disassembly**. (A–D) Effect of AKT family members: BEAS2B cells were transfected with scrambled control siRNA, siAKT1, siAKT2 or siAKT3, then treated with DMSO or MLN4924 (0.3 μmol/L) under cilia initiation condition for indicated periods of time, followed by western blotting with indicated Abs (A) or immunofluoresence staining of cilia and photography (B). Percentage of ciliated cells were counted (C) or cilia length measured (D), the results plotted. Shown is mean ± SEM from three independent experiments. **P < 0.01, ***P < 0.001. (E**–**H) Effect of knockdown of AKT family members on cilia disassembly and growth: BEAS2B cells were transfected with scrambled control siRNA, siAKT1, siAKT2 or siAKT3, then treated with DMSO or MLN4924 (0.3 μmol/L) under cilia disassembly condition for 2 h, followed by Western blotting with indicated Abs (E) or immunofluoresence staining of cilia and photography (F). Percentage of ciliated cells were counted (G) or cilia length measured (H), the results plotted. Shown is mean ± SEM from three independent experiments. **P < 0.01

In mammalian cells, the AKT kinase family has three isoforms: AKT1, AKT2 and AKT3. Although being encoded by three distinct genes, they share a high degree of identity at amino acid levels with overall similar functions (Wang et al., 2017). However, several studies showed that different AKT isoforms could also execute specific functions in a manner dependent of cellular context and substrate specificity (Polytarchou et al., 2011). To determine whether AKT regulation on ciliogenesis is AKT1 specific, we used siRNA knockdown approach and directly compared the effect of three AKT isoforms on inhibitory effect of MLN4924 under both cilia assembly and disassembly culture conditions. The siRNA-based knockdown was effective against AKT1 and AKT2, but less against AKT3 (Fig. 4A). While AKT1 knockdown stimulated ciliogenesis when acting alone, and fully rescued MLN4924 inhibitory effect in combination, knockdown of AKT2 or AKT3, in contrast, further inhibited ciliogenesis either acting alone or in combination with MLN4924 with more robust seen in AKT3 knockdown (Fig. 4B and 4C). Furthermore, unlike AKT1 knockdown which prolonged ciliary length, knockdown of neither AKT2 nor AKT3 had any effect (Fig. 4D).

We next determined the knockdown effect of three isoforms under cilia disassembly condition (Fig. 4E), and found a rescue effect on percentage of ciliated cells and ciliary length only by AKT1, not by AKT2 or AKT3 (Fig. 4F–H). Collectively, among AKT family isoforms, AKT1 is only one that specifically regulates cilia initiation and cilia growth (judged by ciliary length) under both cilia assembly and disassembly conditions.

We further determined potential involvement of few well-known ciliary regulatory proteins in mediating siAKT1 effect. While AKT2 knockdown had no effect on the levels of CP110, Cep97 or NDE1, a centrosomal phosphoprotein and a negative regulator of ciliary length (Kim et al., 2011), knockdown of AKT1 or AKT3 reduced the levels of Cep97, and interestingly, AKT1 knockdown specifically reduced NDE1 levels (Fig. S4A–C). Given that AKT1 and AKT3 had opposite effect on cilia initiation and growth, it is unlikely that Cep97 would play a role in the process. We, therefore, focused on NDE1 and determined its potential role in mediating siAKT1 effect by a rescue experiment. Ectopic expression of NDE1, to much higher levels than that of endogenous one (Fig. S4D), had minor effect at earlier (6 h) but no effect at later time points (12 h and 24 h) on cilia initiation without affecting ciliary length, when in combination with AKT1 knockdown (Fig. S4E and S4F). Thus, AKT1 regulation of cilia initiation or ciliary growth/length is independent of NDE1, or CP110 and Cep97.

### AKT1 specifically regulates cilia length through pAKT1-Thr^308^

**Figure 5:**
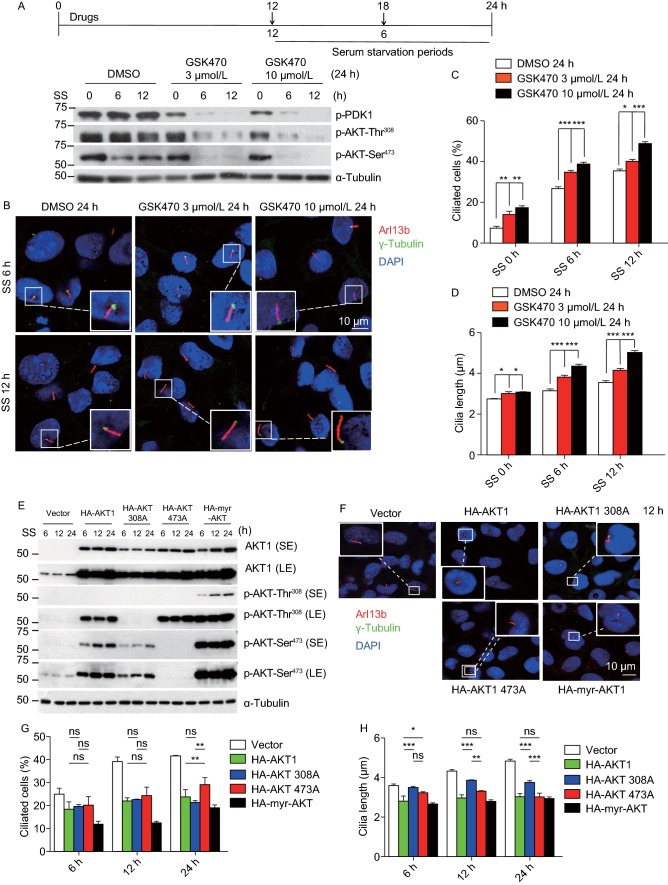
**pAKT-Thr**^**308**^**is responsible for cilia growth**. (A–D) Blockage of AKT1 activation promotes cilia initiation and growth: BEAS2B cells were pretreated with GSK470 (3 μmol/L or 10 μmol/L) for 12 or 18 h prior to serum starvation for 6 or 12 h, respectively (A, top panel), followed by Western blotting with indicated Abs (A, bottom panel) or immunofluoresence staining of cilia and photography (B). Percentage of ciliated cells were counted (C) or cilia length measured (D), the results plotted. Shown is mean ± SEM from three independent experiments. *P < 0.01, **P < 0.01, ***P < 0.001. (E–H) pAKT1-Ser^473^ blocks cilia formation, whereas pAKT1-Thr^308^ blocks cilia growth: BEAS2B cells were transfected with various plasmids expressing wild type AKT1 (HA-AKT1), or mutants, HA-AKT1-308A, HA-AKT1-473A or HA-myr-AKT1, followed by serum-starvation for indicated periods of time, and Western blotting with indicated Abs (E) or immunofluoresence staining of cilia and photography (F). Percentage of ciliated cells were counted (G) or cilia length measured (H), and the results plotted. Shown is mean ± SEM from three independent experiments. *P < 0.01, **P < 0.01, ***P < 0.001

Given that the main AKT1 kinase activity is mediated through pAKT1-Thr^308^ (Fresno Vara et al., 2004), we then focused on whether AKT1 effect on cilia length is mediated via pAKT1-Thr^308^. We used GSK2334470 (designated as GSK470), a highly selective PDK1 inhibitor (Najafov et al., 2011), which inactivated pAKT1-Thr^308^ as a PDK1 downstream substrate, as well as pAKT1-Ser^473^, known to undergoes auto-phosphorylation in a PDK1-dependent manner (Persad et al., 2001). Cells were pretreated with GSK470 for 12 or 18 h prior to serum starvation for 6 or 12 h, respectively (Fig. 5A, top panel). As expected, GSK470 effectively inactivated AKT1 phosphorylations at both Thr^308^ and Ser^473^ sites (Fig. 5A, bottom panel). Significantly, it promoted ciliogenesis by increasing percentage of ciliated cell population and cilia length in a dose and time dependent manner in BEAS2B cells (Fig. 5B–D). The similar results were also observed in RPE1 cells (Fig. S5A–C). Given that pAKT1-Ser^473^ is involved in regulation of ciliated population only, we proposed that pAKT1-Thr^308^ is likely responsible for the control of cilia length.

To further determine the role of AKT phosphorylation on ciliogenesis, we used the gain-of-function approach. BEAS2B cells were transfected with various plasmids expressing wild-type AKT (HA-AKT1), two phosphorylate mutants (HA-AKT1-308A or HA-AKT1-473A) or AKT constitutively active form (HA-myr-AKT1) (Fig.5E), followed by serum-starvation for various time points. Constitutively active HA-myr-AKT effectively inhibited ciliogenesis, as reflected by reduced ciliated population and shortened cilia length. While AKT1-473A mutant is the least effective in blocking ciliogenesis, AKT1-308A mutant is less effective in blocking cilia length (Fig. 5F–H). Similar results were observed in RPE1 cells (Fig. S5D–G). Taken together, our results support that notion that pAKT1-Ser^473^ plays a major role in blocking ciliogenesis, which is MLN4924-dependent, whereas pAKT1-Thr^308^ plays a major role in blocking cilia length in a MLN4924-independent manner.

### Cross-talk between AKT-1 and pVHL in regulation of ciliogenesis

**Figure 6:**
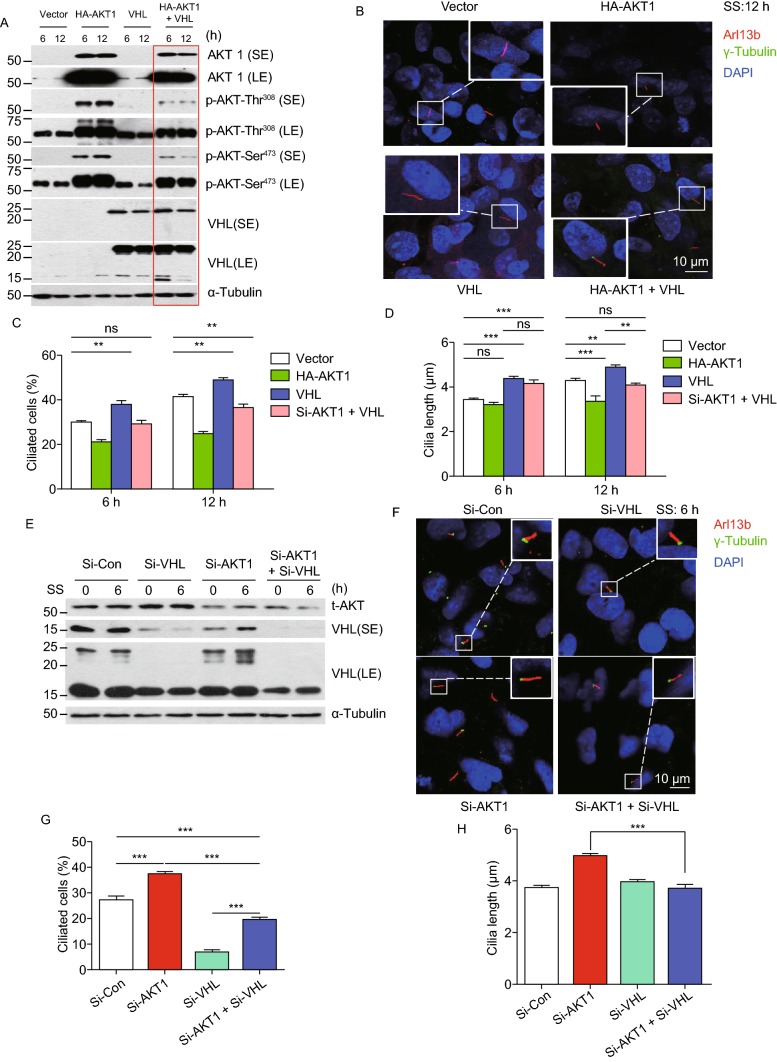
**Cross-talk between AKT1 and pVHL in regulation of ciliogensis**. (A–D) Effect of VHL on AKT level, activity, and ciliogenesis: BEAS2B cells were transfected with vector, HA-AKT1 or VHL alone or in combination, followed by serum starvation for indicated time points, and Western blotting with indicated Abs (A), or immunofluoresence staining of cilia and photography (B). Percentage of ciliated cells were counted (C) or cilia length measured (D), and the results plotted. Shown is mean ± SEM from three independent experiments. **P < 0.01, ***P < 0.001. (E–H) Effect of pVHL knockdown on ciliogenesis triggered by AKT1 knockdown: BEAS2B cells were transfected with scrambled control siRNA, siAKT1, or siVHL alone or in combination, followed by serum starved for 6 h, and Western blotting with indicated Abs (E) or immunofluoresence staining of cilia and photography (F). Percentage of ciliated cells were counted (G) or cilia length measured (H), and the results plotted. Shown is mean ± SEM from three independent experiments. ***P < 0.001

Previous studies have demonstrated that pVHL positively regulates ciliogenesis in a number of cellular systems (Schermer et al., 2006; Kuehn et al., 2007) and pVHL can act either upstream or downstream of AKT1 in a cell context dependent manner (Frew et al., 2008; Guo et al., 2016; Castaneda et al., 2017). We, therefore, determined potential involvement of pVHL in AKT regulation of ciliogenesis. In both BEAS2B and RPE cells, pVHL co-transfection with AKT1 inhibited the levels of total AKT1 as well as two active forms of AKT1, with a greater inhibition on pAKT1-Thr^308^, particularly in RPE cells (Figs. 6A and S6A), and partially rescued AKT1-induced suppression of cilia initiation and cilia growth (Figs. 6B–D and S6B–D). Using a loss-of-function approach, we found that pVHL knockdown, on one hand, inhibited ciliogenesis, and blocked the stimulatory effect of AKT knockdown (Fig. 6E–G). On the other hand, abrogated promoting effect of AKT knockdown, while itself having no effect on cilia growth (Fig. 6H). Similar results on ciliogenesis were seen in RPE1 cells (Fig. S6E–G). Taken together, AKT1 and pVHL cross-talk with each other to precisely regulate cilia initiation and growth.

### MLN4924 inhibits hair regrowth by blocking ciliogenesis in mouse skin

**Figure 7:**
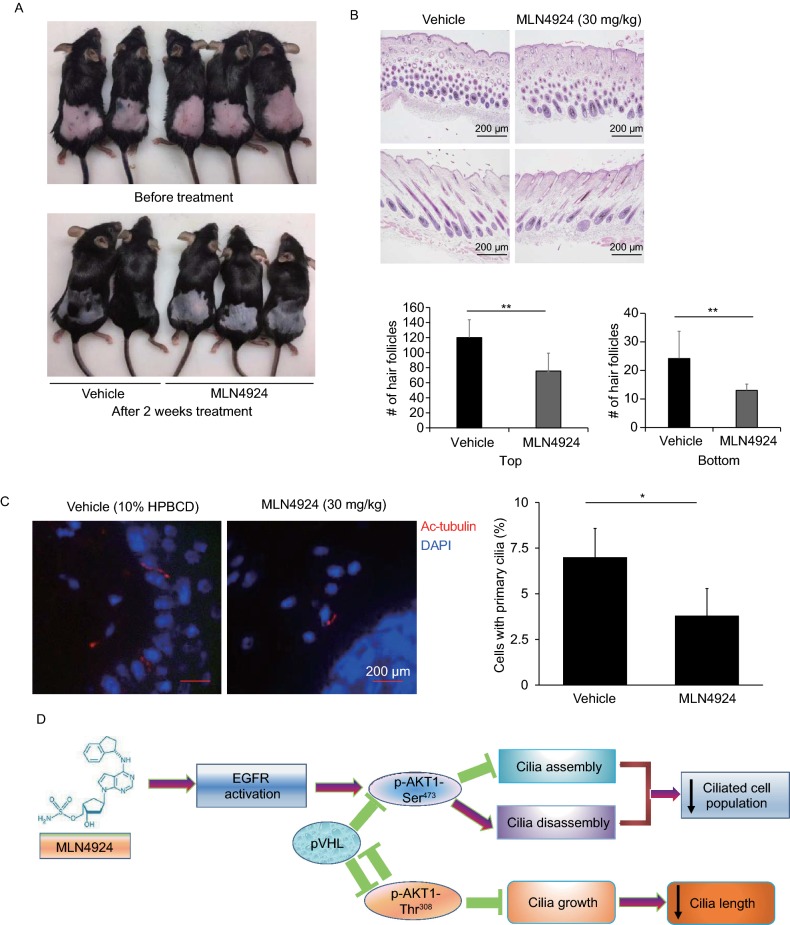
**MLN4924 inhibits hair regrowth by blocking ciliogenesis in mouse skin**. (A) The dorsal hairs of mice were removed on day 1. On day 2, mice were injected with MLN4924 (30 mg/kg) along with vehicle control, once a day, 5 days per week for 2 weeks. The photo images were taken before and after 2 weeks of treatment. (B and C) Mice skin were harvested at the end of 2-week treatment, fixed, embedded, sectioned, stained with H&E and photographed. The hair follicles were counted and result blotted (B), or skin sections were stained with immonofluorescence using ciliary marker acetylated tubulin (red), positive cells were counted. All scale bars are 200 µm. Shown is mean ± SEM from randomly selected 5 skin areas harvested from at least two mice. (D) The working model. See text for details

Finally, we extended our cell culture study to an *in vivo* mouse model. It is well-established that hair regrowth requires primary cilia (Lehman et al., 2009; Goetz and Anderson, 2010; Rishikaysh et al., 2014; Abe and Tanaka, 2017). To determine *in vivo* consequence of MLN4924 inhibition of ciliogenesis, we depilated mouse hair on dorsal areas and divided mouse into two groups: one received the vehicle control and the other receiving non-toxic dose (30 mg/kg) of MLN4924 via s.c. injection, 5 days per week for two weeks (Wei et al., 2012). The hair regrowth was recorded at the end of two-week treatment. As shown in Fig. 7A, MLN4924 administration significantly inhibited hair regrowth. The H&E staining of skin tissue sectioned from depilated areas showed a significantly reduction of skin follicle in MLN4924-treated skin (Fig. 7B). Reduced number of cilia positive hair follicles was also observed in MLN4924-treated group (Fig. 7C). We have repeated this experiment with MLN4924 being applied either locally or via s.c injection and found that suppression of cilia regrowth was only effective via s.c. injection (Fig. S7). Taken together, our study showed that cilia inhibitory effect MLN4924 can be extended from cell culture setting to an *in vivo* mouse model.

## DISCUSSION

MLN4924, also known as pevonedistat, is the first in class small molecular inhibitor of neddylation activating enzyme, currently at the Phase I/II clinical trial for anti-cancer application, acting alone or in combination with other chemo-drugs (Zhao et al., 2014; Zhou et al., 2018). In preclinical settings, MLN4924, via blocking the entire neddylation modification, showed numerous anti-cancer activities by inducing growth arrest, apoptosis, autophagy, inhibiting angiogenesis and inflammatory responses in a cell context dependent manner (for review, see (Zhou et al., 2018)). In this study, we made an unexpected finding that MLN4924 blocked primary ciliogenesis by inhibiting initiation and promoting disassembly without affecting cilia growth or cilia length. Importantly, this inhibition is a general event, seen in multiple cell lines, and occurs rapidly in a manner independent of G_1_/G_0_ arrest, a status known to promote ciliogenesis (Ishikawa and Marshall, 2011).

It is previously unknown that neddylation modification could directly regulate the process of ciliogenesis, although a number of ciliary-associated proteins (e.g., CP110 and Aurora A) are substrates of Cullin-RING ligases (CRLs) (D’Angiolella et al., 2010; Hasanov et al., 2017), whose activity required NEDD8 attachment via neddylation (Deshaies et al., 2010). Nevertheless, the involvement of these substrates was excluded in our experimental settings via siRNA knockdown approaches. We then turned our attention to the EGFR-AKT axis, based upon our recent finding that MLN4924 at nanomolecular concentration, triggered EGFR dimerization to promote stem cell characteristics under serum starved condition (Zhou et al., 2016), a condition that triggers ciliogenesis as well (Pugacheva et al., 2007). Indeed, inactivation of either EGFR or AKT1 completely abrogated the inhibitory effect of MLN on ciliogenesis, indicating a causal role.

In the process of using siRNA knockdown of AKT1 to rescue MLN4924 inhibitory effect, we made yet another unexpected finding that knockdown of AKT1, but not its family members AKT2 or AKT3, not only restored ciliated cell population up to the control level, a process which is MLN4924-dependent, but also stimulated cilia growth by extending its length in MLN4924 independent manner. The length of cilia is precisely regulated in cells, and abnormal cilia length correlates with several ciliopathies (Nigg and Raff, 2009). Previous studies have shown that cilia length is subject to regulation by several proteins, including NDE1, F-actin, VHL, FBXW7, AKT1 (Kim et al., 2011; Dere et al., 2015; Nikonova and Golemis, 2015; Suizu et al., 2016; Zhang et al., 2016). We found that under our experimental setting, cilia length is not regulated by NDE1, but by AKT1, particularly pAKT1-Thr^308^ and pVHL. Two lines of evidence support a role of pAKT1-Thr^308^. First, GSK2334470, a selective inhibitor of PDK1, which blocks pAKT1-Thr^308^, elongated cilia in a dose-dependent manner; Second, ectopic expression of AKT1-T308A, a AKT1-Thr^308^ dead mutant, inhibits cilia growth to a much lesser extent than that of wild-type AKT1 or AKT-Ser^473^ (Fig. 5H). It is worth noting that a previous study using the RPE1 or MEF cells showed that knockdown or knockout of AKT1/2, particularly AKT2, suppressed cilia development by reducing ciliated cell population and cilia length (Suizu et al., 2016). We used human BEAS2B cells and found that AKT2 knockdown reduced ciliated cell population without affecting cilia length, whereas AKT1 knockdown significantly increased ciliated population and cilia length (Fig. 4), suggesting a cell line dependent effect of AKT family members on cilia development. We reason our finding by proposing that AKT1 knockdown would suppress cell growth and survival, allowing cells to differentiation in a way to trigger cilia development under optimal serum starvation condition. Nevertheless, exact underlying mechanism, by which AKT1 regulates cilia development in a cell context dependent manner, remains elusive, but certainly an interesting subject for future investigation.

VHL has been widely implicated in regulation of ciliogenesis (Kuehn et al., 2007), which acts as either upstream or downstream regulator of AKT1 in a cell context dependent manner (Thoma et al., 2007; Guo et al., 2016). In our experimental setting in both BEAS2B and RPE1 cells, ectopic VHL co-expression inactivated AKT1 phosphorylation, particularly on pAKT1-Thr^308^. Functionally, VHL expression increased ciliated cell population and promoted cilia growth, it also fully rescued negative effect of AKT1. The pVHL knockdown, on the other hand, reduced ciliated cell population, but not affected cilia growth. pVHL knockdown also fully rescued stimulating effect of siAKT1 for both cilia initiation and cilia growth. These results clearly suggested that pVHL acts upstream of AKT1 as a positive regulator of cilia formation and growth. Under the condition of AKT1 knockdown, when cilia formation and growth were stimulated, pVHL appears to act downstream of AKT1 to mediate its effect which is abrogated by simultaneous pVHL knockdown.

Finally, we extended our cell culture-based observations to an *in vivo* mouse model in which re-growth of depilated skin hair requires primary cilia (Lehman et al., 2009; Goetz and Anderson, 2010; Rishikaysh et al., 2014; Abe and Tanaka, 2017). Indeed, MLN4924 treatment significantly inhibited ciliated cell population in affected mouse skin with reduced hair follicles, indicating effectiveness of MLN4924 in blocking *in vivo* ciliogenesis. Abnormal cilia overgrowth was observed in a number of human cancers such as adenocarcinoma of the lung and colon, follicular lymphoma and a subtype of medulloblastoma (Han et al., 2009; Yasar et al., 2017). By blocking ciliogenesis, MLN4924 may have a unique application to treat human cancers which rely on cilia for growth or drug resistance (Jenks et al., 2018).

In summary, our study supports a working model as follows. a) MLN4924 triggers EGFR dimerization, leading to activation of pAKT1-Ser^473^ to inhibit ciliogenesis by blocking cilia assembly and promoting cilia disassembly. b) Activation of pAKT1-Thr^308^ via the PI3K-PDK1 signal inhibits cilia growth and shortens cilia length. pVHL acts either upstream or downstream of AKT1 to positively regulate ciliogenesis (Fig. 7D). Our finding that AKT1 negatively regulates ciliogenesis may have a therapeutic implication. In human diseases with ciliopathies, such as polycystic kidney (4), the selective AKT1 inhibitor may have a unique application to promote cilia formation.

## MATERIALS AND METHODS

### Cell lines and chemicals

BEASB, cells were purchased from ATCC. The RPE1 and IMCD3 cell lines were the gift from Dr. Qing Zhong. BEAS2B cells were cultured in RPMI 1640 medium (Invitrogen, Carlsbad, CA). IMCD3 cells were cultured in DMEM medium (Invitrogen). RPE1 cells were cultured in F12 medium (Invitrogen). All media were supplemented with 10% fetal bovine serum. MLN4924 and MK2206 were purchased from ApexBio (Houston, TX), GSK2334470 from Selleckchem (Houston, TX). Erlotinib was a gift from Dr. Wanhong Xu. All compounds were dissolved in dimethyl sulfoxide (DMSO) and stored at −20 °C.

### Immunofluorescence microscopy and imaging analysis

Cells were subjected to serum starvation in the absence or presence of DMSO, or MLN4924 (0.3 μmol/L), MK (1 μmol/L), Erlotinib (10 μmol/L) and GSK470 (3&10 μmol/L) for various periods of time. Cilia were visualized by an indirect immunofluorenscence staining, as previously described (Pugacheva et al., 2007). Image acquisition was performed using a Nikon A1X60 microscope system (Nikon A1-Ti, Tokyo, Japan), equipped with NIS-elements software (Nikon A1-Ti). Percentage of ciliated cells and cilia length were counted and measured. Cilia length was measured using ImageJ software (NIH). The results were plotted in a bar graph. Shown is mean ± SEM from three independent experiments.

### Immunoblotting (IB)

Cell lysates after various treatments were prepared and subjected to IB analysis as described (Gu et al., 2007). The sources of primary antibodies were as follows: Cullin-1 (Santa Cruz, CA), phospho-AKT-Ser473, phosphor-AKT-Thr308, pan-AKT, AKT1, AKT2, AKT3, Aurora A, VHL, Phospho-Aurora A (Thr288)/Aurora B (Thr232)/Aurora C (Thr198) (D13A11), PDK1, phospho-PDK1-Ser^241^ and Rictor (53A2) (Cell Signaling, Denver, CO), γ-tubulin, α-tubulin Clone AA13, acetylated tubulin Clone 6-11B-1, β-actin (Sigma, St. Louis, MO), CP110, NDE1, Cep97, Arl13B (Proteintech, Wuhan, P.R.C). UBA3 (Abcam, Shanghai, P.R.C). EGFR and phospho-EGFR-Tyr845 were gifts from Dr. Wanhong Xu,

### FACS (fluorescence-activated cell sorting) analysis

Cells post various treatments were harvested and fixed in 70% ethanol overnight. Cells were washed twice with ice-cold phosphate buffered saline (PBS) and then stained with propidiumiodide (PI, 20 mg/mL, Sigma) solution for 30 min in the dark. The samples were then analyzed using a BD FACScan flow cytometer facility (Franklin Lakes, NJ) for cell cycle distributions.

### SiRNAs, plasmids and transfection

The siRNA oligoes to knockdown *AKT1*, *AKT2*, *AKT3*, *CP110*, *Cep97* or *VHL* were synthesized by GenePharma (Shanghai, P.R.C). The sequences of siRNAs are as follows: *AKT1*: 5′-GCGTGGTGAATACATCAAGACTT-3′; *AKT2*: 5′-GCGTGGTGAATACATCAAGACTT-3, *AKT3*: 5′-TAGTCCAACTTCACAAATTGC-3; CP110: 5′-AAGCAGCATGAGTATGCCAGT-3′; *CEP97*: 5′-GATGAGAAGTGAAATCAATTT-3′; *VHL*: 5′-TATCACACTGCCAGTGTATACCTC-3′. The siRNA oligoes to knockdown *UBA3* was synthesized by RIBOBIO (Guangzhou, P.R.C). The sequence of si-UBA3 is 5′-GCTTCTCTGCAAATGAAAT-3′. The plasmid expression constructs, encoding *HA-AKT1*, *HA-AKT1-308A*, *HA-AKT1-473A or HA-myr-AKT*, were gifts from Dr. Wenyi Wei. Cells were transfected with siRNA duplexes (10 nmol/L) or plasmids using Lipofectamine RNAiMAX or Lipofectamine 3000, respectively, according to the manufacturer’s instructions (Life Technologies, Carlsbad, CA).

### Animal studies

All animal studies were conducted in accordance with the guidelines established by the University of Michigan Committee on Use and Care of Animals. The C57BL/6 mice at 8 weeks of age were used. Dorsal hair was removed by a hair removal cream, Nair, as described (Wang et al., 2013). In the following day, mice were injected s.c. with MLN4924 (30 mg/kg, five times per week), along with the HPBCD vehicle control (Wei et al., 2012), for two weeks, or locally applied with MLN4924 (0.3 μmol/L in 70% ethanol) once a day, 5 days per week for 2 weeks. Mice were then photographed and sacrificed. The dorsal skin biospies were harvested, fixed in cold 4% paraformaldehyde overnight and embedded in paraffin. Skin sections were stained with H&E and examined under microscope. Immonofluorescence staining was performed with antibodies against acetylated tubulin and γ-tubulin and counterstained with DAPI.

### Statistical analysis

Data with two groups were analyzed by two-tailed Student’s *t*-tests, and data with multiple groups were analyzed by one-way ANOVA, followed by Bonferroni post hoc test using GraphPad Prism statistical programs (GraphPad Prism, San Diego, CA). Results were expressed as mean ± SEM from three independent assays. The *P* < 0.05 was considered statistically significant.

## Electronic supplementary material

Below is the link to the electronic supplementary material.

Supplementary material 1 (PDF 3774 kb)
