# Supplementary material for: Neddylation inhibitor MLN4924 suppresses cilia formation by modulating AKT1
Source: Protein Cell. 2019 Mar 9;10(10):726–44. doi: 10.1007/s13238-019-0614-3 (PMC6776484; doi:10.1007/s13238-019-0614-3)
Supplement: Supplementary file 1 — Supplementary material 1 (PDF 3774 kb) [file 13238_2019_614_MOESM1_ESM.pdf]

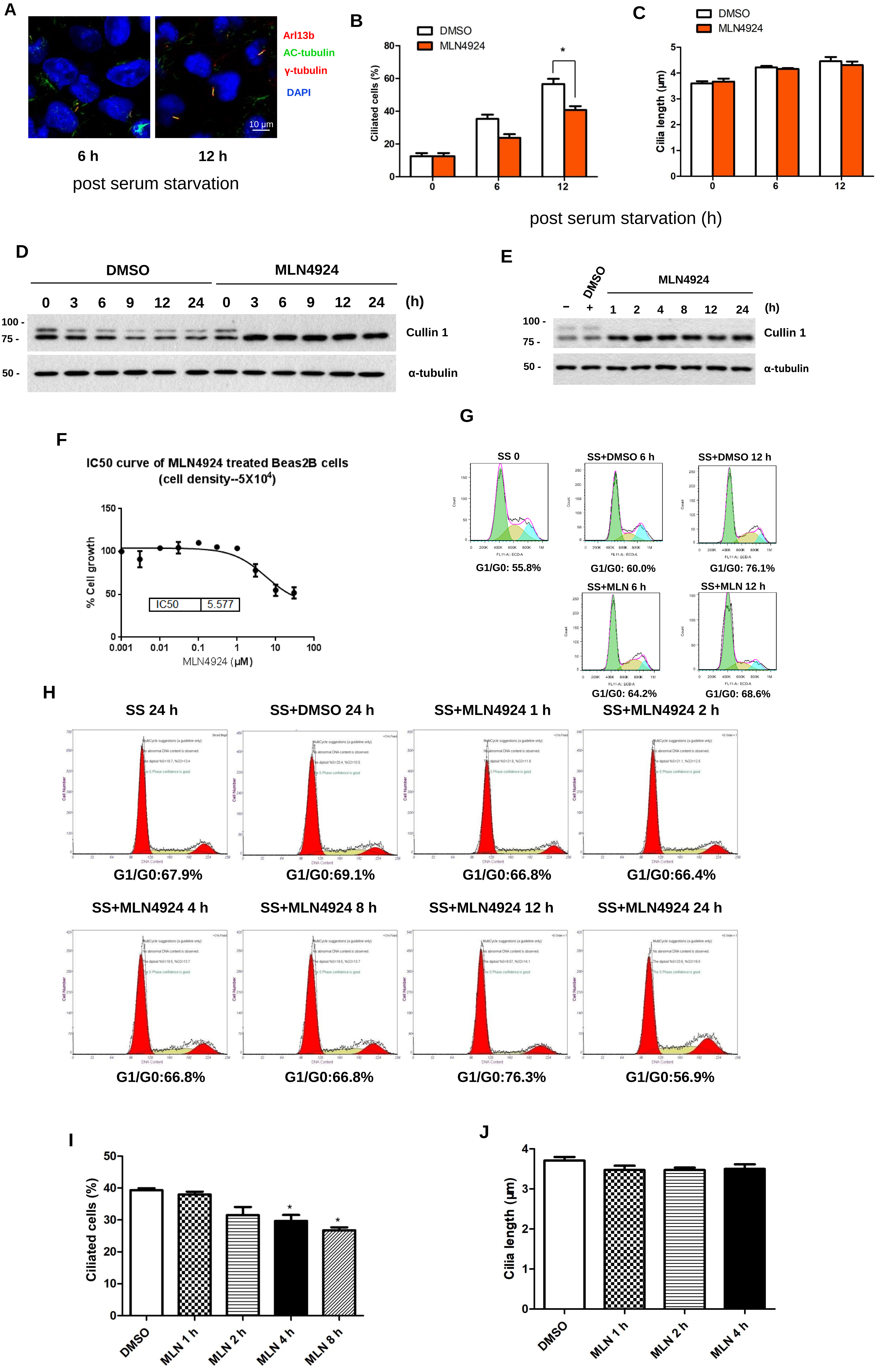

Figure S1. MLN4924 inhibits ciliogenesis by inhibiting cilia assembly and promoting cilia disassembly in multiple cell lines without G0/G1 phase change.

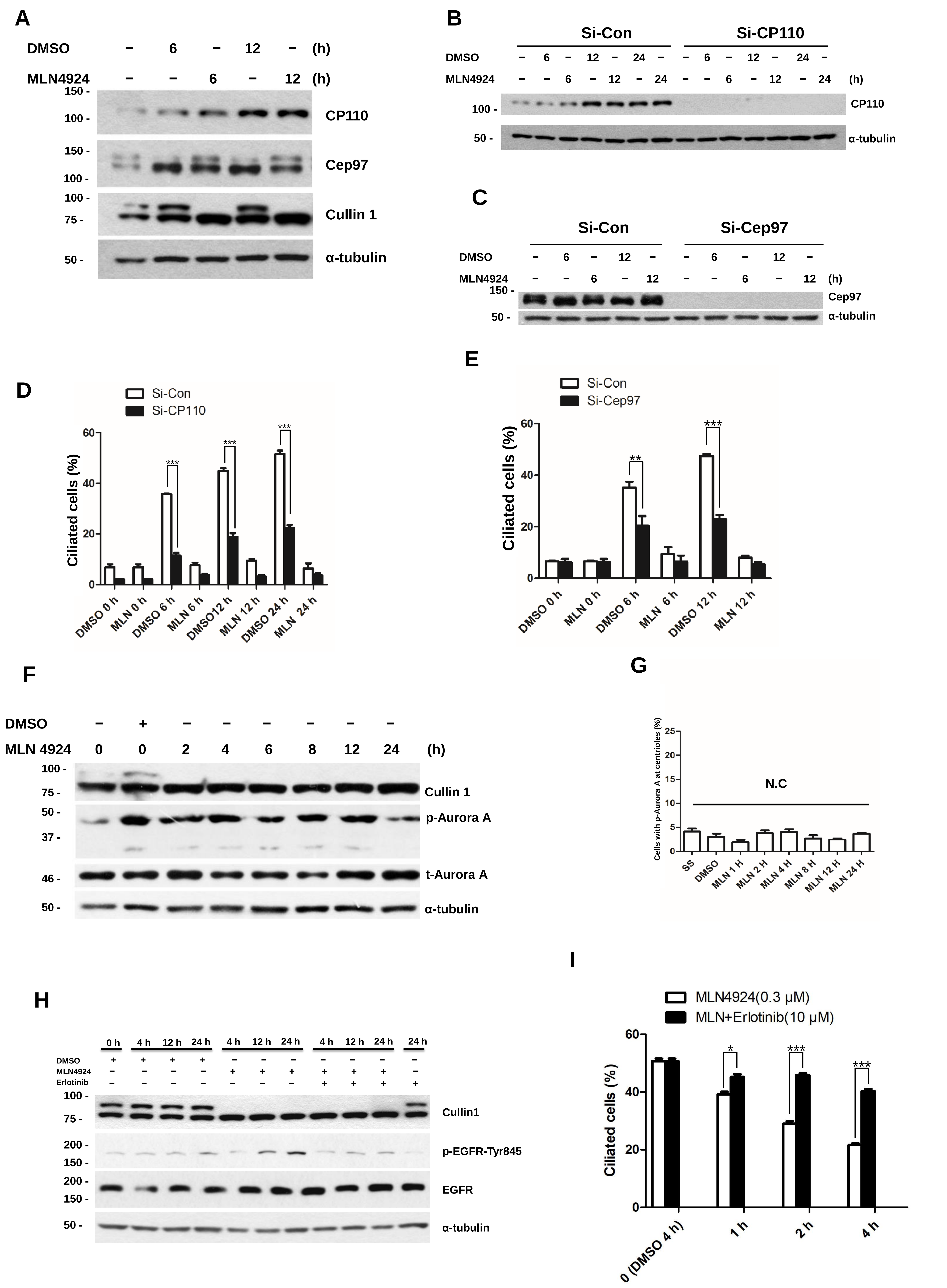

Figure S2. MLN4924 inhibition of cilia assembly is independent of CP110 and Cep97, but dependent of EGFR.

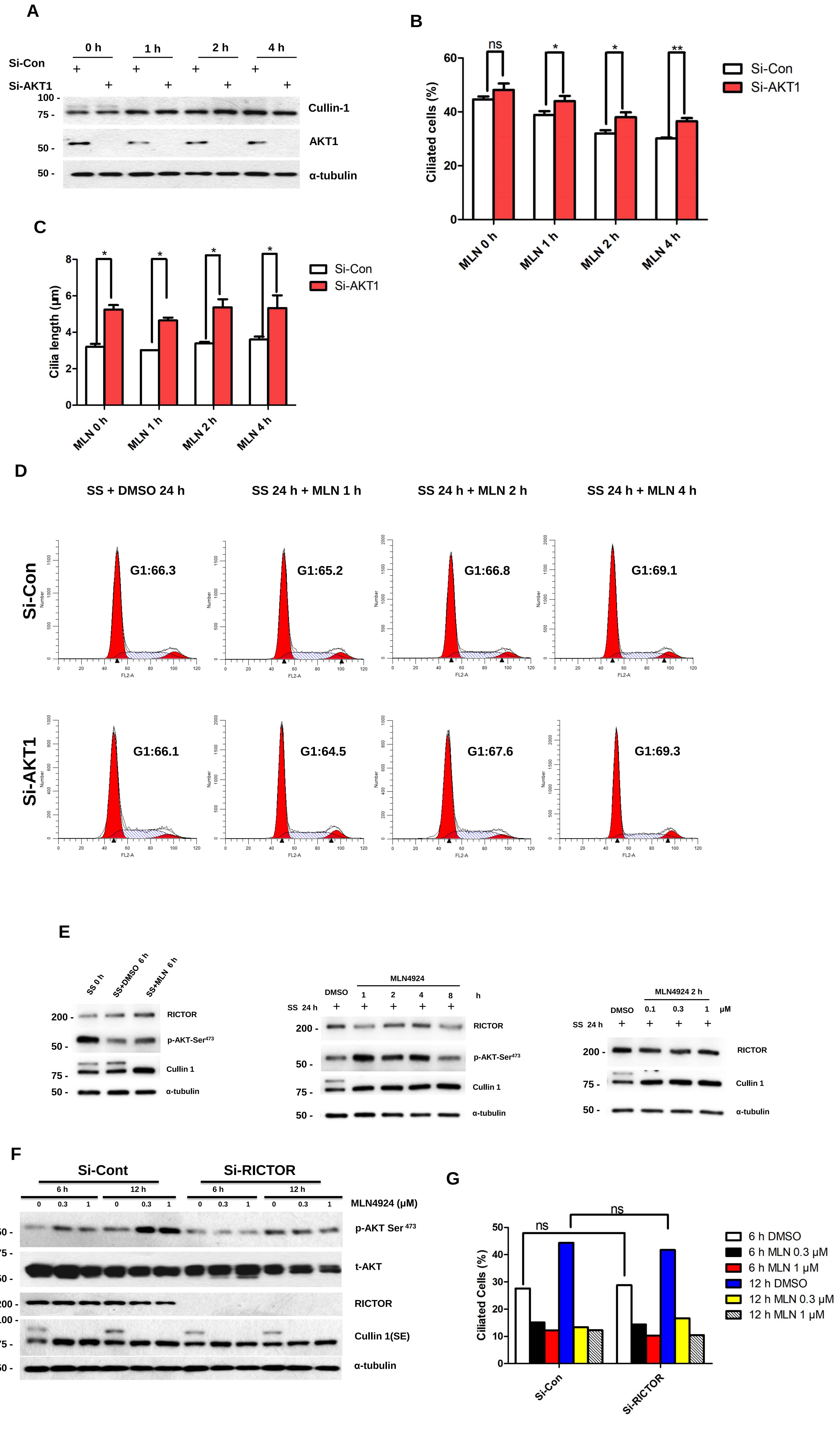

Figure S3. AKT1 involvement in MLN4924-induced cilia disassembly in RPE1 cells.

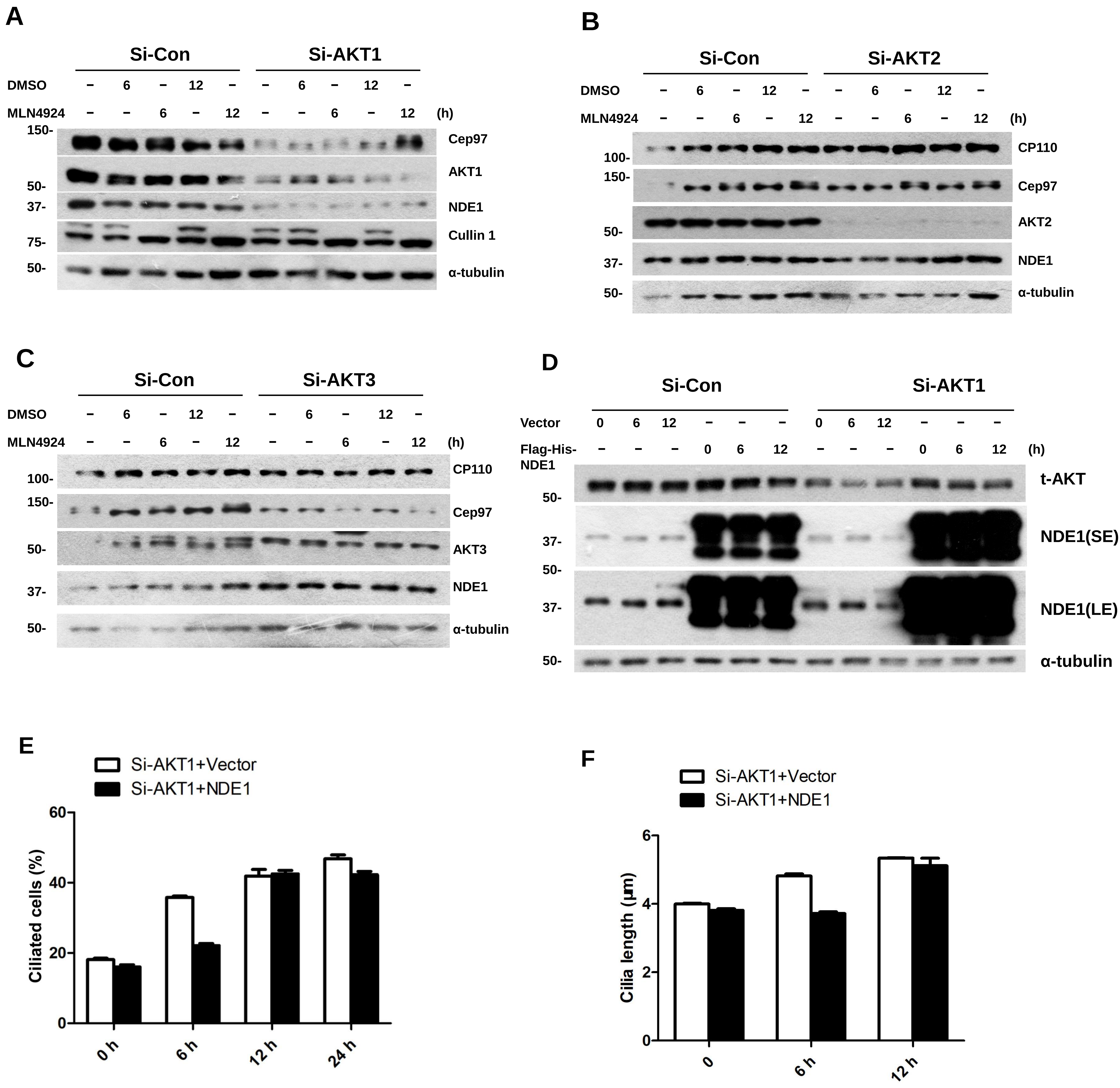

Figure S4. Effect of AKT family members and NDE1 on ciliogenesis.

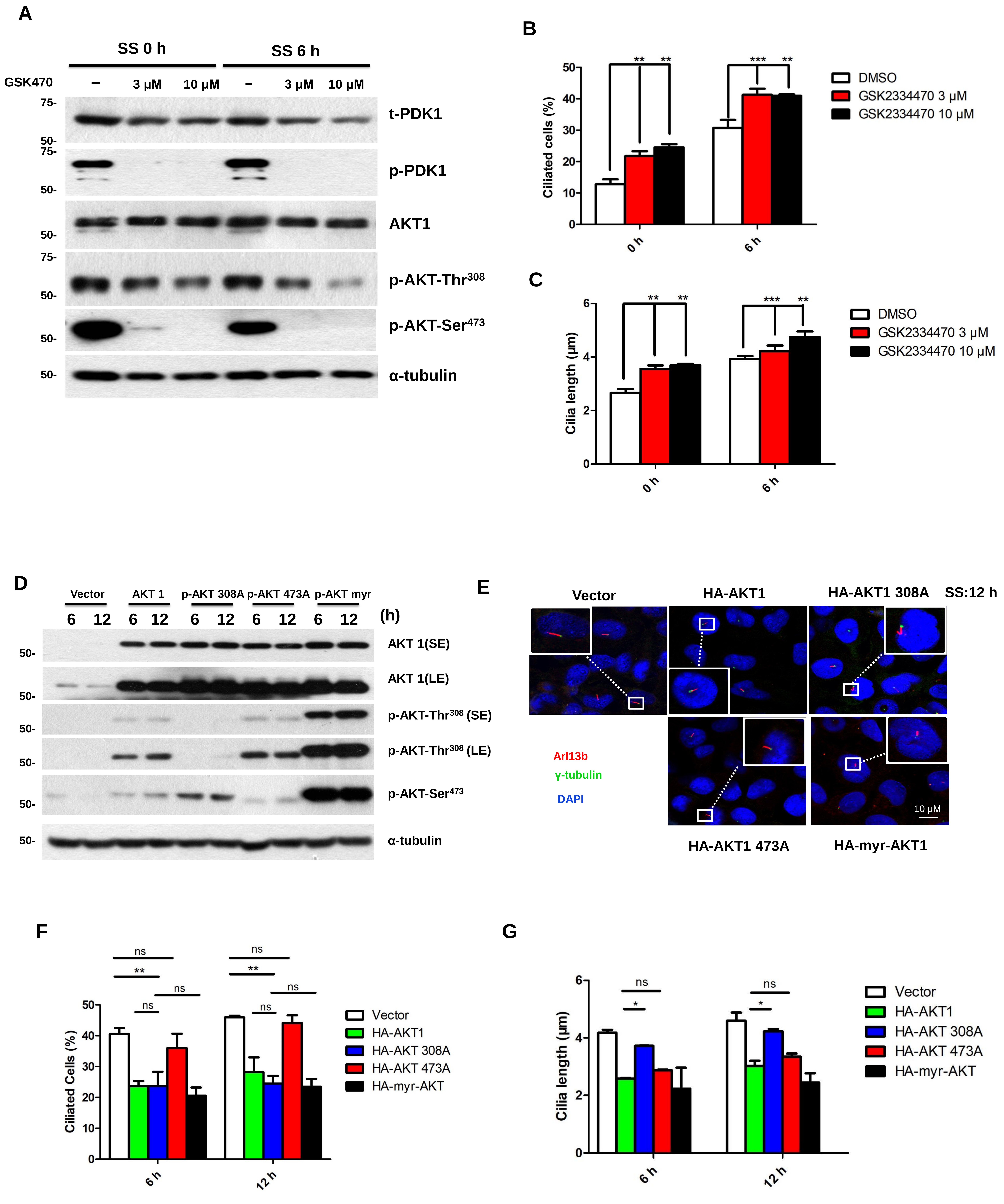

Figure S5. pAKT-Thr^{308} is responsible for inhibition of cilia growth in RPE1 cells.

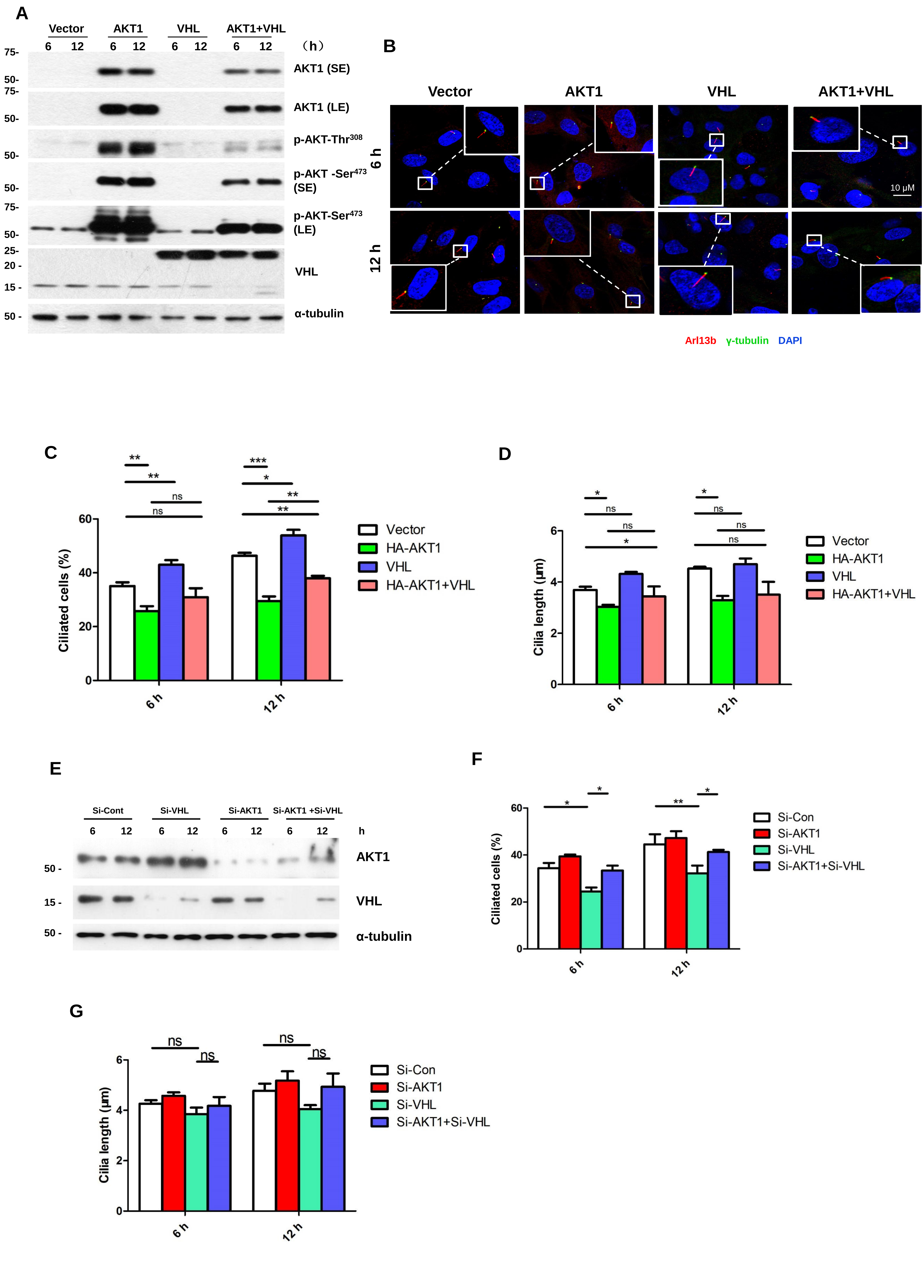

Figure S6. Cross-talk between AKT1 and pVHL in regulation of ciliogensis in RPE1 cells.

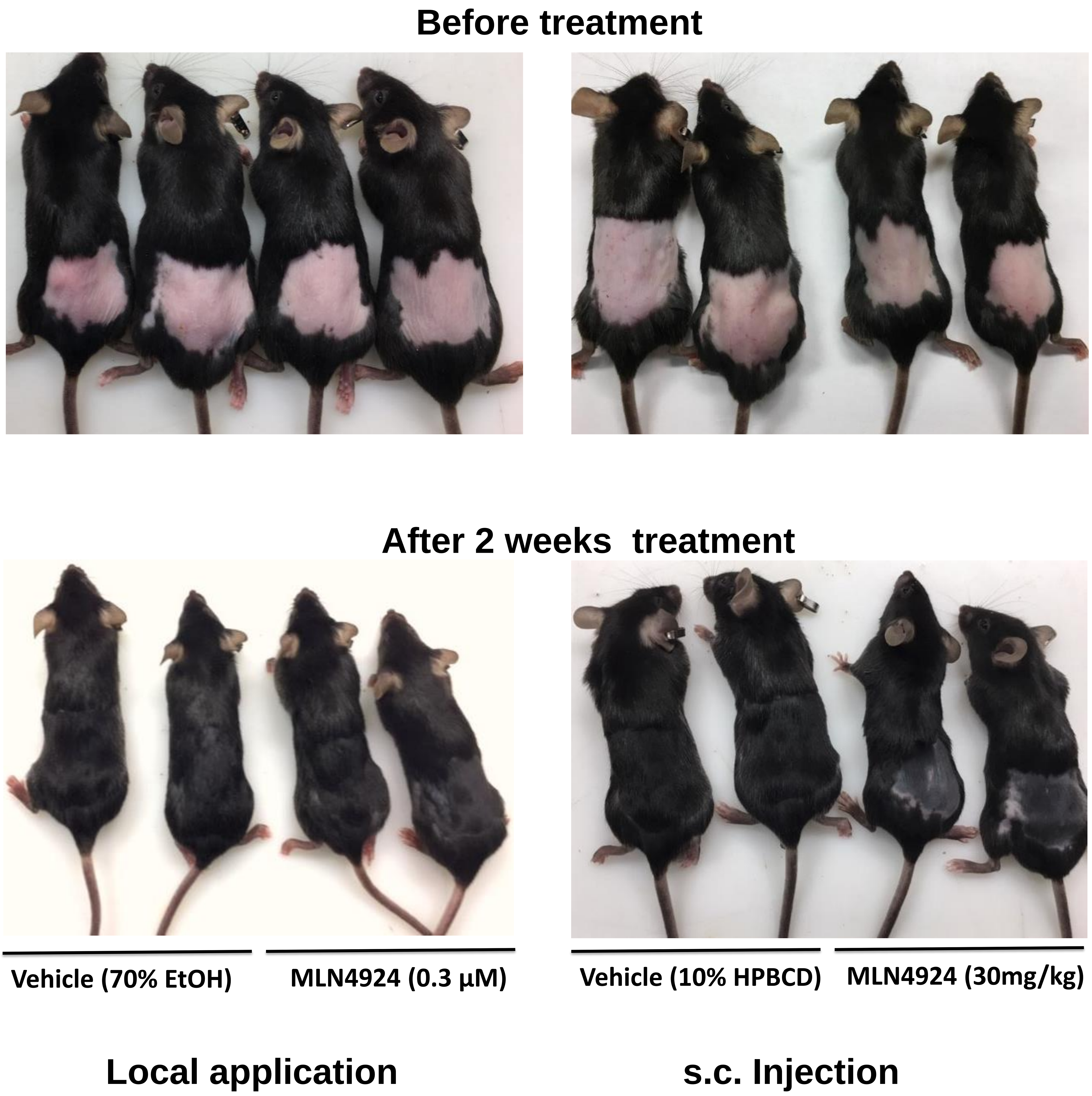

Figure S7. MLN4924 suppresses hair regrowth.
